# Time, space and social interactions: exit mechanisms for the Covid-19 epidemics

**DOI:** 10.1038/s41598-020-70631-9

**Published:** 2020-08-13

**Authors:** Antonio Scala, Andrea Flori, Alessandro Spelta, Emanuele Brugnoli, Matteo Cinelli, Walter Quattrociocchi, Fabio Pammolli

**Affiliations:** 1 Applico Lab, CNR-ISC, Rome, Italy; 2 Big Data in Health Society, Rome, Italy; 3 Gubkin Russian State University of Oil and Gas, Leninsky Prospekt, Moscow, Russia; 4 Impact, Department of Management, Economics and Industrial Engineering, Politecnico Di Milano, Milan, Italy; 5 Univ. Di Pavia, Pavia, Italy; 6 Center for Analysis Decisions and Society, Human Technopole and Politecnico Di Milano, Milan, Italy; 7 Univ. Di Venezia ’Ca Foscari, Venezia, Italy

**Keywords:** Information theory and computation, Nonlinear phenomena

## Abstract

We develop a minimalist compartmental model to study the impact of mobility restrictions in Italy during the Covid-19 outbreak. We show that, while an early lockdown shifts the contagion in time, beyond a critical value of lockdown strength the epidemic tends to restart after lifting the restrictions. We characterize the relative importance of different lockdown lifting schemes by accounting for two fundamental sources of heterogeneity, i.e. geography and demography. First, we consider Italian Regions as separate administrative entities, in which social interactions between age classes occur. We show that, due to the sparsity of the inter-Regional mobility matrix, once started, the epidemic spreading tends to develop independently across areas, justifying the adoption of mobility restrictions targeted to individual Regions or clusters of Regions. Second, we show that social contacts between members of different age classes play a fundamental role and that interventions which target local behaviours and take into account the age structure of the population can provide a significant contribution to mitigate the epidemic spreading. Our model aims to provide a general framework, and it highlights the relevance of some key parameters on non-pharmaceutical interventions to contain the contagion.

## Introduction

The spread of Covid-19 has induced the introduction of a large variety of epidemic models, aimed to identify specific mechanisms relevant for policy design^1^. Although mathematical models contribute to generate relevant information both on the diffusion of the virus and on the socio-economic consequences of the epidemics^2,3^, scientific uncertainties are still high, and available data do not sustain neither univocal evaluations of the proposed policies nor exact predictions of the potential future outcomes^4^.

To contain the Covid-19 epidemic, governments worldwide have adopted severe social distancing policies, ranging from partial to total population lockdown interventions^5^. As a consequence, policy restrictions have led to a sudden stop of economic activities in many sectors, since the portion of the population most affected by Covid-19 infections has proven to be the age class of active people between 15 and 64 years^6,7^. Overall, the impact of contagion and lockdown measures on health and economic activities results substantial and pervasive^8–10^.

Against that background, we introduce a model-based scenario analysis for Covid-19 diffusion in Italy and we highlight how geographical and demographic dimensions influence the epidemic spreading and the effects of lockdown solutions, while providing some general indications on relevant exit mechanisms^9^. The general behavior of our framework holds for the vast class of epidemic models where the transmission rate is proportional to the number of susceptible people times the density of infected ones, thus being relevant both for deterministic and stochastic models as long as the initial fluctuation regime is overcame^11^.

We focus on the determinants of short-term interventions in response to an emerging epidemic, when geographical and demographic dimensions are included in the model. Our goal is general in nature, since we focus on two relevant decomposability conditions under which partial dynamics influence the overall configuration of the system under investigation^12–15^. Specifically, we study how (i) mobility restriction interventions and (ii) the timing of the lockdown lift, jointly affect the total fraction of infected people, the peak prevalence, and the delay of the epidemic dynamics. Our analysis reveals two fundamental sources of heterogeneity in the diffusion process: geographical boundaries and age classes^9^. Finally, we show how these dimensions can shape policy interventions aiming at containing the epidemic outbreak.

This paper contributes to the extant literature on trade-offs between mitigation, i.e. slowing down the epidemic contagion, and suppression, i.e. temporarily compressing the risk of contagion^9,16–18^. Notwithstanding micro data on individual profiles are not taken into account in our compartmental model, we show how the inclusion of geographical and age classes uncovers relevant features impacting on model results on virus diffusion, providing some guidance to policy makers.

We show that an early lockdown shifts the epidemic in time and that beyond a critical threshold of the intensity of the lockdown, the epidemic would tend to fully recover its strength as soon as the lockdown is lifted. As a consequence, specific mitigation strategies for a second wave must be prepared during the lockdown phase. To provide some guidance on the relative importance of different general strategies, we first study how the heterogeneity of the intensity of mobility flows across Italian administrative Regions influences the observed delays of the contagion. The relative strength of intra-Regional mobility with respect to inter-Regional mobility flows implies that, once the epidemic has started, it then tends to develop independently within each Region, as also empirically observed in simulations of Covid-19 spread in China^19^. Then, we study the impact of patterns of interaction within and between age classes, finding that the structure of the interactions is of primarily importance in estimating post-lockdown effects. According to our results, age-based mitigation strategies, which can affect local behaviours can represent a key ingredient to contain a second wave and to protect age-classes with higher incidence of severe cases.

## A minimalist framework for Covid-19 diffusion in Italy

To analyze mobility-restriction policies, we introduce a minimalist compartmental model^17,20^. Although a variety of models, mechanistic, statistic and stochastic^21–25^, have been proposed to study the spread of diseases and, recently, of Covid-19^19,26–30^, scientific uncertainty is high. In order to ensure that their output is informative, calibration must be grounded on reliable data not suffering from the lack of homogeneous procedures, such as in medical testing, sampling or collection^18,31^. Not to mention the difficulty to assess the impact of variability in social habits during the epidemic^17,32^. Moreover, especially in the early phases of the epidemic—i.e. the ones characterised by an exponential growth—different models sharing a given reproduction number ${R}_{0}$ fit the data with equivalent accuracy (see “Methods” for a detailed discussion).

For these reasons, since our aim is to focus on some fundamental qualitative scenarios of epidemic dynamics and not on detailed predictions, we keep the transmission scheme as simple as possible, to avoid confounding effects. In so doing, we rely on a variant of the $SIR$ model. The $SIR$ framework has been widely applied to model flu-like epidemic through a basic formulation which implies a population of $N$ individuals divided into three states: Susceptible $S$, Infective $I$, and Removed $R$, where removed indicates individuals who are either recovered from the disease and immune to further infection, or dead. Infective individuals may have contacts with randomly chosen individuals of all states at an average rate $\beta$ per unit time, and recover and acquire immunity, or die, at an average rate $\gamma$ per unit time. If those whom infective individuals have contact with are in the susceptible state, then they become infected.

We adapt the $SIR$ framework to the observed data reported by the Italian National Health Institute (ISS), taking into account the number of infected people and the ones with observable symptoms, the mobility flows among Italian municipalities and the social mixing structure of the population. Differently from the $SIR$, our $SIOR$ model relies on four compartments. Hence, $S$(usceptible) individuals can become $I$(nfective) when meeting an infective individual, while $I$(nfectives) either become $O$(bserved)—i.e. individuals who present symptoms acute enough to be detected from the national healthcare system—or are $R$(emoved) from the infection cycle; $O$(bserved) individuals switch into the $R$(emoved) class either because they recovered or died. Notice that the introduction of the additional compartment $O$ gives us the possibility of adjusting our model’s parameters with respect to the observed data, since this class of “observable” individuals refers to the fraction of infected people detected by the national healthcare system. Moreover, we are implicitly absorbing the number of deaths in the $R$(emoved) compartment of the model; thus $R$ comprises both the recovered people (who are likely to be not susceptible anymore) and the small fraction of those who do not overcome the disease. Finally, since it is not yet clear the role of the asymptomatic phase^33,34^, in the model we implicitly assume that asymptomatic individuals are infective and we assume that their removal time is the same of the $I$ class.

The model is described by the following differential equations, while its graphical representation is presented in Fig S1 of the SI:

1 $$\begin{array}{ll}{\partial }_{t}S& =-\beta S \frac{I}{N}\\ {\partial }_{t}I& =\beta S \frac{I}{N}-\gamma I\\ {\partial }_{t}O& =\rho \gamma I-hO\\ {\partial }_{t}R& =(1-\rho )\gamma I+hO\end{array}$$

where $N=S+I+O+R$ is the total number of individuals in the population, the transmission coefficient $\beta$ is the rate at which a $S$usceptible becomes infected upon meeting an $I$nfected individual, and $\gamma$ is the rate at which an $I$nfected either becomes $O$bserved or $R$emoved from the infection cycle. The additional parameters of the $SIOR$ model are $\rho$, the fraction of infected that become observed from the national healthcare system, and $h$, the rate at which observed individuals are removed from the infection cycle. Notice that we consider $O$(bserved) individuals not infecting others, being strictly isolated (see “Methods” for baseline parameters’ calibration strategy). As for the $SIR$ model, the basic reproduction number can be calculated as ${R}_{0}=\beta /\gamma$ and the stationary state can be estimate as follows: let us consider $X=O+R$, it holds that ${\partial }_{X}S=-{R}_{0}S$ and $S(t\to \infty )=N{e}^{-{R}_{0}X(t\to \infty )}$; then, since $O(t\to \infty )=I(t\to \infty )=0$, it follows that $R(t\to \infty )=N-S(t\to \infty )$ and we recover the same solution of the $SIR$ model: $S(t\to \infty )=N{e}^{-{R}_{0} [N-S(\to \infty )]}$.

The Italian lockdown measures of March 8th–9th, 2020^35,36^ aimed to change mobility patterns and to reduce the intensity of social contacts, both through quarantine measures and by increasing awareness of the importance of social distancing. We assume the rate $\gamma$, which relates to the “medical” evolution of the disease, is not affected by lockdown, which instead is intended as a non-pharmaceutical intervention to prevent the epidemic spreading. Analogous arguments apply to the rates $h$ and $\rho$ related to the observable compartment (although $\rho$ could be influenced by different testing schemes or alert thresholds). On the other hand, the transmission coefficient $\beta$ can be thought as the product $C\lambda$ of a contact rate $C$ times a disease-dependent transmission probability $\lambda$. Hence, if we assume that the speed of Covid-19 mutation is irrelevant on our timescales, lockdown strategies mostly influence $\beta$ by reducing the contact rate $C$ between individuals.

Without loss of generality, we assume that, after the lockdown day ${t}_{\mathrm{Lock}}=15$ (corresponding to the 9th of March), the contact rate drops down by a factor $\alpha$ and hence $\beta \to \alpha \beta$. By fitting the observed data ${Y}^{\mathrm{Obs}}$ for a symmetric period of $15$ days after ${t}_{\mathrm{Lock}}$, and by performing a bootstrap sensitivity analysis, we find $\alpha =0.49\pm 0.01$, i.e. a reduction of $\sim 50{\%}$ in infectivity and hence in ${R}_{0}$. Our analysis is in line with the observed reduction in ${R}_{0}$ in response to combined non-pharmaceutical interventions which has been shown to be on average around 64% compared to the pre-intervention values across several countries^37^.

Table 1 shows the value of the model parameters. Notice that $\beta =0.35\;\text{day}^{-1}$ corresponds to a basic reproduction number ${R}_{0}=3.5$ in line with the average ${R}_{0}$ found in Ref.^38^. Moreover, since patients needing hospitalization and intensive care represent the highest burden for healthcare facilities, in the figures of the paper we adopt this value estimated as 3.5% of the total patients, as reported by ISS^7^, instead of $I$.

**Table 1:** Parameters used for the $SIOR$ model.

Model parameters		
$\beta =0.35\;\text{day}^{-1}$	$\gamma =1/10\;\text{day}^{-1}$	$h=1/9\;\text{day}^{-1}$
${t}_{0}=-30\;\text{days}$	$\rho =0.40$	$\alpha =0.49$

To evaluate the impact of lockdown measures on mobility, we extensively analyze data set on Facebook (FB) aggregated mobility flows; those data are part of the Facebook project “Data for Good”, and illustrate mobility patterns of FB users, who allowed the social network to track their location^39,40^. The data set accounts for daily movements of approximately 4 M individuals in a period of 1 month, from February 24th to March 24th.

The Facebook mobility data show a reduction in mobility of 15% at Regional level and of 73% at inter-Regional level during the lockdown phase; however, as we will point out later, mobility has a strong impact at the beginning stage of the epidemic in each single Region/country, while it has much lower effects on its evolution with each territory.

**Figure 1:**
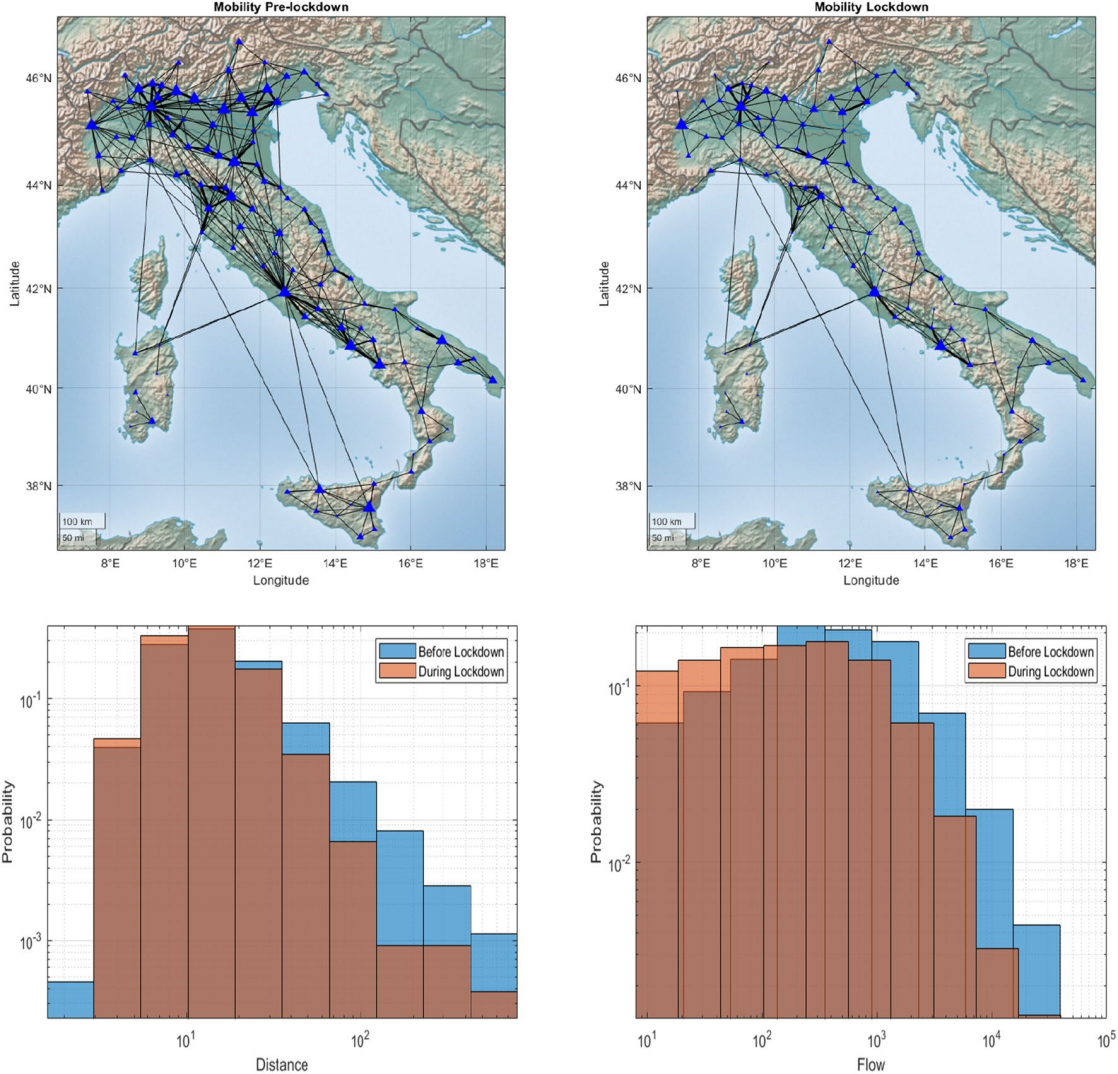
Mobility patterns in Italy before and during the lockdown. The upper panels show national mobility flows (at district level) before and during the lockdown phase. The size of nodes and edges is proportional to the intra- and inter-district mobility, respectively. The map has been created using Matlab 2019b (see https://it.mathworks.com/). Lower panels report the distributions of mobility patterns in Italy before and during the lockdown. The right panel shows the reduction of the travelled distance (in km) and left panel reports the number of trips before the intervention (blue) and during the lockdown phase (orange).

To characterize the impact of non-pharmaceutical interventions in Italy, we analyze the FB network of mobility at the province level of detail. The upper panels of Fig. 1 present the evolution of the Italian network of mobility before and during the national lockdown. This network representation has been obtained by building an averaged graph over a window of 14 days before and during national lockdown, i.e., each edge has a weight which is the average of all the observations in such period. While the main mobility hubs such as, Turin, Milan, Bologna, Rome, and Naples, continue to be connected during the lockdown phase, the Italian peninsula exhibits a mobility flows network which is severely affected by national lockdown restrictions. As shown by the lower panels of Fig. 1, the deployment of the lockdown has also reduced both the travelled distance and the flow of travelling people.

Since we are interested in the factors which influence the exit dynamics from lockdown, and not in the accurate quantitative predictions of specific strategies, we rely on scenario analysis, where the lockdown is abruptly lifted and the system is allowed to return to the pre-lockdown parameters’ configuration. Such an approach clearly describes a worst-case estimate of the intensity of the second wave of contagion at the end of the lockdown. We consider several simplified scenarios, where we use the $SIOR$ model described by System (1) with the parameters presented in Table 1. First, we analyze the relationships between the post-lockdown dynamics and the restrictions implemented by the national authorities. Second, we modify the framework by introducing a metapopulation $SIOR$ model to study the effect of explicitly considering Italy as a collection of separate administrative entities (Regions). Finally, we consider the effects of social interactions across age classes.

**Figure 2:**
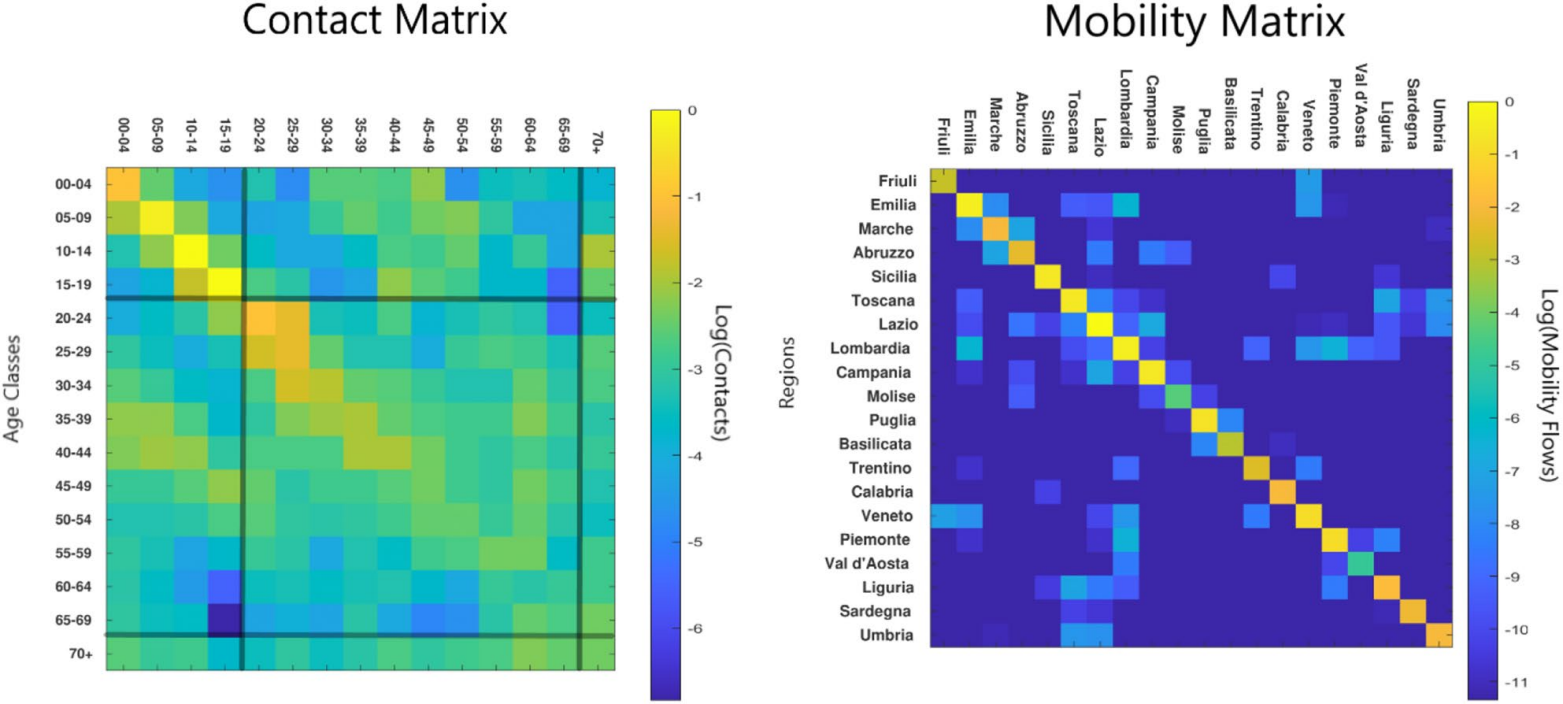
Left Panel: social contact matrix (from Ref.^41^). Right panel: inter-Regional mobility matrix (from Facebook project^39^ “Data for Good”). The intensity of the colorbar indicates the strength of a matrix element (light colors: high values; dark colors: low values). The inter-age social mixing matrix is dense; hence age classes dynamics are strongly coupled. The inter-Regional mobility flows are very sparse (i.e. off diagonal elements are order of magnitude lower than diagonal elements). This means that most of the people move within the same Region of origin; hence, the dynamics of different Regions can be considered as “almost” decoupled.

Interestingly, mobility flows^40^ and inter-age social mixing^41^ display opposite features. In fact, on the one hand, the social contact matrix is dense (Fig. 2, left panel), indicating that age classes dynamics are strongly coupled. On the other hand, the inter-Regional mobility matrix is very sparse (Fig. 2, right panel), indicating that Regions have their own independent dynamics.

## National scenarios and exit mechanisms

We first consider a simple exit strategy consisting in lifting the lockdown at a time ${t}_{\mathrm{Unlock}}$ after the peak of $O$ has occurred. We assume that the infection proceeds uncontrolled up to time ${t}_{\mathrm{Lock}}$ when lockdown restrictions are put in place for the period $[{t}_{\mathrm{Lock}},{t}_{\mathrm{Unlock}}]$. During the lockdown phase, we assume that the transmission coefficient $\beta$ is reduced by a factor $\alpha$; finally, we let the parameter $\beta$ to turn back to its initial value. Hence, such scenario does not consider other policy interventions, like forcing social distances and the use of personal protection devices like masks, which may contribute to contain the contagion.

**Figure 3:**
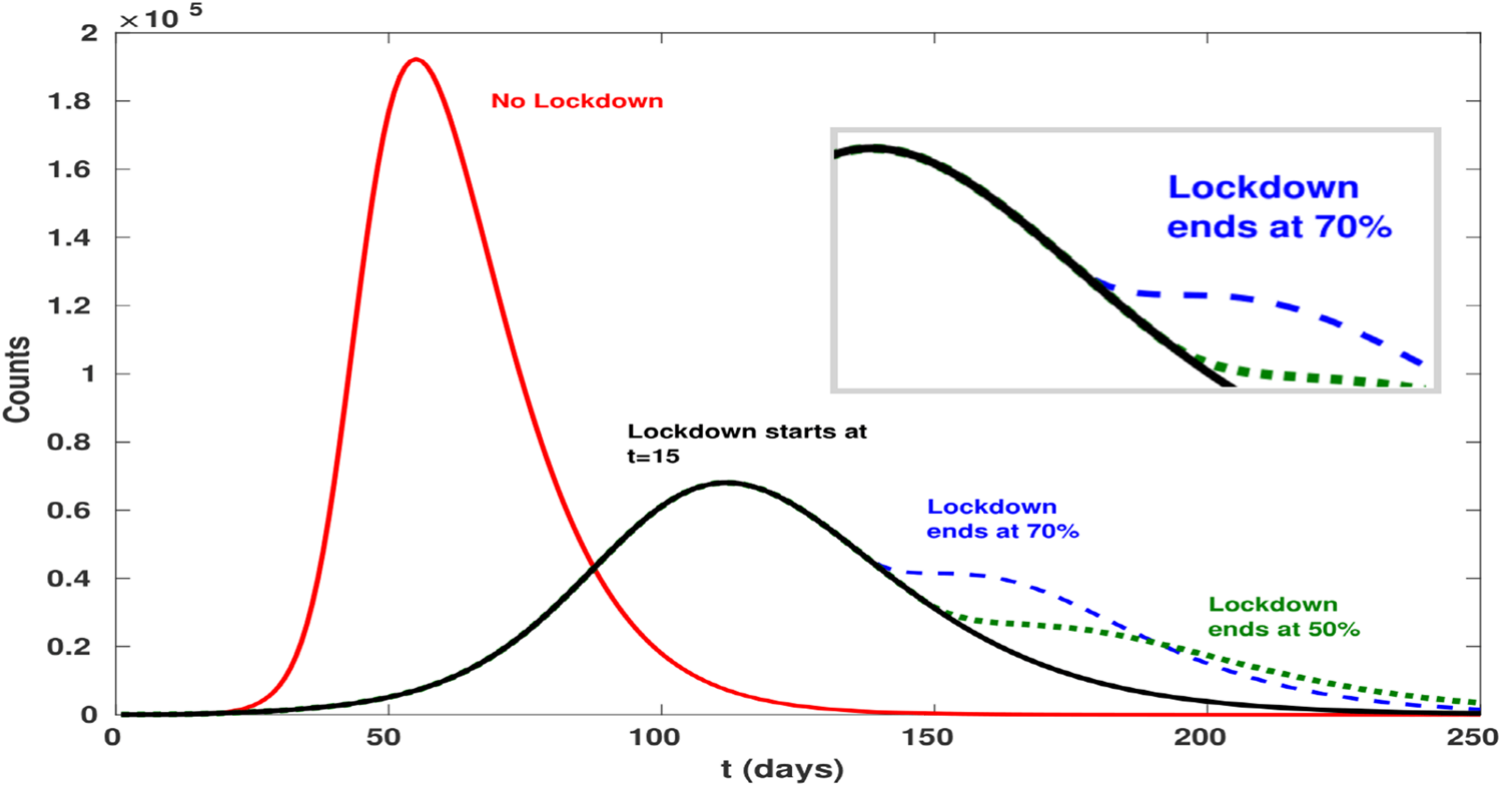
Comparison of the scenarios where the lockdown is relaxed after the percentage of people with visible symptoms ($O$) is reached the 70% and the 50% of the reported cases peak. Lifting the lockdown earlier makes the epidemic disappear faster, but has higher impact on the number of hospitalized and intensive care patients; moreover, lifting the lockdown too early can result in a rebound of the number of cases, as shown in the inset.

Our results show that lockdown lowers the peak of $O$—i.e. the individuals with observable symptoms—to $\sim 70{\%}$ with respect to the free epidemic case, but it also doubles the time of its occurrence from $\sim 1.9$ to $\sim 3.8$ months, thus constituting an extremely obnoxious effect for the sustainability conditions of the economy of a country. However, since the number of hospitalized patients and—most importantly—the number of patients in intensive care is only a fraction of $O$, lowering the peak puts less stress on the healthcare system. In an ideal framework, lifting the lockdown when the number of infected people per unit time $\beta S(t)I(t)/N$ is lower than the average number of recovering people $\gamma I(t)$ would ensure that the number of infections would continue to decrease. In real life, on the other hand, the set-up is more fuzzy: given the lack of information, governments might decide to resort on some heuristics, such as lifting the lockdown once the observed people $O$ have dropped to a suitable percentage of the maximum peak. As an example, after $\sim 4.7$ months the peak would have reduced to 70% of its initial value, while after $\sim 5.2$ months to 50%. Notice that, the earlier the lockdown is lifted, the faster $O$ decays to zero even if it starts from higher figures and could possibly induce a second wave of epidemic spreading. All such effects are shown in Fig. 3 and in Fig. S3 of SI.

Our framework sustains the identification of several mechanisms. The first is related to the timeliness of the lockdown, i.e. to the choice of anticipating ${t}_{\mathrm{Lock}}$. As expected, anticipating the lockdown (i.e. well before the infection peak of the uncontrolled epidemic) reduces the height of the peak at the cost of both delaying its appearance and widening the duration of the epidemic. Conversely, lifting the lockdown too soon can make epidemic to start again by reaching values higher than the ones observed before the release. A peculiar and counter-intuitive effect can be generated if the lockdown is anticipated too much: in fact, a too early lockdown may pose the risk of delaying the start of the epidemic without attenuating its severity.

In addition, increasing the strength $\alpha$ of the lockdown not only delays the time at which it could be lifted, but it also induces a stronger re-start of the epidemic in the post-lockdown phase. This may trigger the need for new closures, with repeated lockdown phases that would obviously be unsustainable in terms of social and economic costs. Specifically, by increasing the strength $\alpha$ of the lockdown, i.e., the ratio between the transmission $\beta$ after and before the lockdown, the epidemic peak is pushed forward but its height is lower. On the other hand, beyond a critical threshold ${\alpha }_{\mathrm{crit}}$, the number of $R$ecovered would not grow enough ($R\ll N$), while $I$nfected would quickly decrease to zero; hence, the epidemic would tend to fully recover its strength after the lockdown lifting, since it would start from a state $S\sim N$, $R\sim 0$ where the growth of $I$ is again exponential. For instance, in Fig. S3 in SI, we show the impact of lifting the lockdown when the peak is fallen by 30%, noticing that a stronger lockdown induces a more consistent upswing of the epidemic. An analogous effect can be observed by varying the lockdown starting date: anticipating the lockdown ameliorates the epidemic peak by decreasing its height, but pushes it forward and delays the end of the epidemic.

Contrary to what could be naively expected, an early imposition of the lockdown does not ameliorate the epidemic: in fact, anticipating too much the lockdown just shifts the timing of the epidemic, leaving its evolution largely unchanged (see Fig. S4 in SI). This is to be expected every time extreme measures of social distancing are applied in the very early, exponentially growing, stage of the epidemic. In fact, let us consider two countries $A$ and $B$ that have the same population, contact matrix, and number of infected people. If $A$ and $B$ decide to establish a lockdown of strength $\alpha$ at time ${t}_{A}$ and ${t}_{B}$, respectively, then at certain time $t$ any quantity $y$ of the model would have grown as ${y}_{A}(t)\sim {y}^{0} {e}^{{R}_{0}{t}_{A}} {e}^{\alpha {R}_{0}(t-{t}_{A})}$ and as ${y}_{B}(t)\sim {y}^{0} {e}^{{R}_{0}{t}_{B}} {e}^{\alpha {R}_{0}(t-{t}_{B})}$. If there exists a time $t{^{\prime}}$ such that ${y}_{A}(t)={y}_{B}(t{^{\prime}})$, then the epidemic in $A$ and in $B$ will proceed in parallel (even in the non-linear phase) with a delay $t{^{\prime}}-t$. Therefore, if both the epidemic dynamics of $A$ and $B$ are still well approximated by exponential curves at times $t<max\{t,t{^{\prime}}\}$, then $t{^{\prime}}-t\propto -({t}_{A}-{t}_{B})$, i.e., the country that has started earlier the lockdown will experience the same epidemic of the other country, but delayed in time. In particular, for identical initial conditions, we have that:

2 $$t-t{^{\prime}}=-\frac{1+\alpha }{\alpha }\left({t}_{A}-{t}_{B}\right)$$

as long as all the times refer to a period before the end of the initial exponential regime. Such an estimate can be very useful for countries where the epidemic has not yet started. Indeed, calibrating on one own normalized growth curve the time of the lockdown and its strength would give an idea of how long one can delay the full start of the epidemic dynamics.

An additional counter-intuitive mechanism can be considered. Since an attenuation of $\alpha$ corresponds to an effective reproduction number ${R}_{0}^{\mathrm{eff}}=\alpha {R}_{0}$, at the critical value ${\alpha }_{\mathrm{crit}}=1/{R}_{0}$ the epidemic is expected to stay in a quiescent state where it neither grows nor decreases; to be precise, the decrease becomes sub-exponential, thus taking a practically infinite time when the size of the population is large. Thus, after ${t}_{\mathrm{Lock}}$ the system stays stationary until the lockdown is lifted at ${t}_{\mathrm{Unlock}}$; at this point, the epidemic starts growing again as it occurred before the lockdown. In general, if $\alpha <{\alpha }_{\mathrm{crit}}$, the epidemic loses strength but as soon as the lockdown is lifted, it starts again to reach its full strength (see Fig. S5 in SI). Our estimate for the Italian lockdown are $\alpha \sim 0.5>{\alpha }_{\mathrm{crit}}\sim 0.3$. Hence, at least in Regions where epidemic had an early start, it will not be necessary to follow a repeated seek-and-release strategy in the post-lockdown phase. On the contrary, if it can be attained a lockdown strength $\alpha \sim {\alpha }_{\mathrm{crit}}$ without disrupting the economy, the epidemic could be contained until the creation, production and distribution of a vaccine.

## Regional scenarios

**Figure 4:**
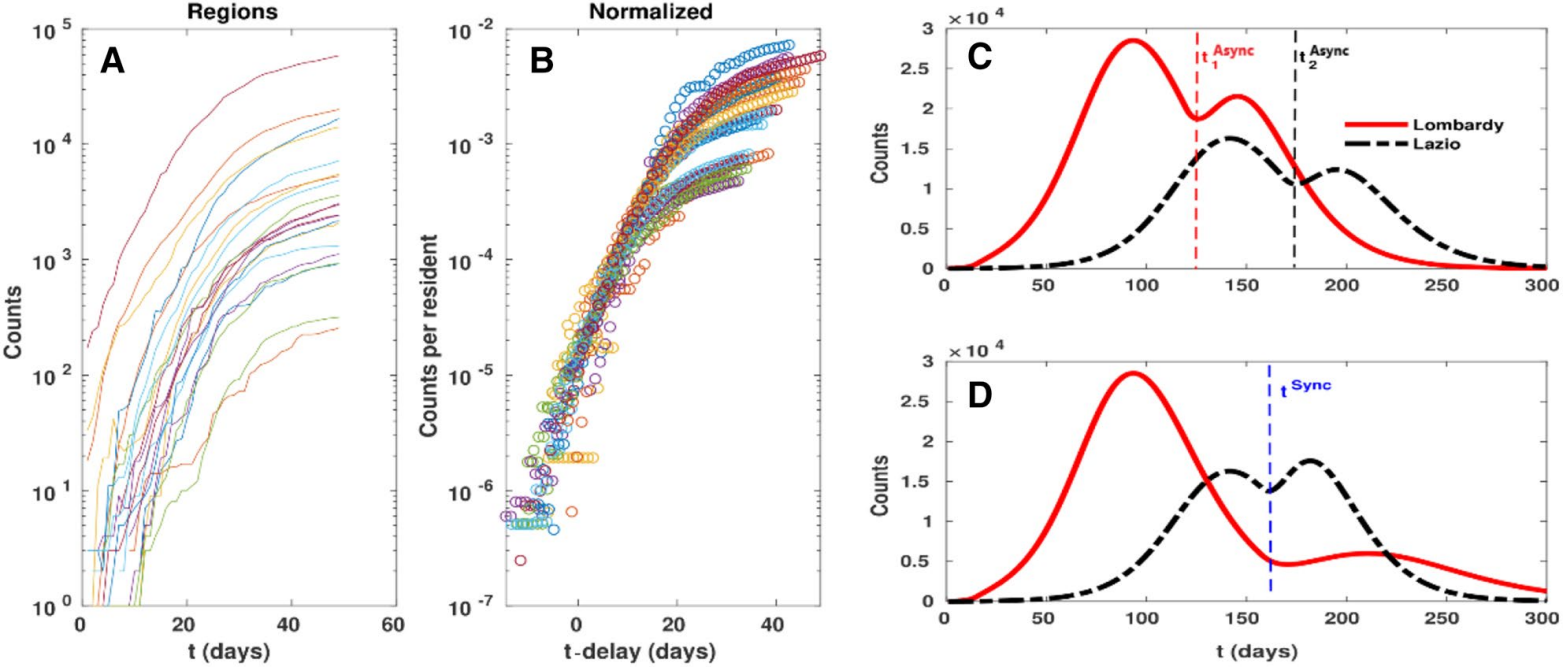
(**A**,**B**) analysis of time delays among the start of epidemic in different Regions (see Table 2). If the dynamics is similar in different Regions, the normalised curves are supposed to be just time shifted versions; (**B**) shows that indeed Regional curves have a good collapse when shifted by their delays. (**C**,**D**) simulated dynamics of an *Async*(hronous) exit strategy (i.e. each Region lifts the lockdown following its own policy) with respect to a *Sync*(hronous) exit strategy (i.e. the lockdown lift follows the same policy, but applied to a nation wide scale). In particular, ${t}^{Sync}$ corresponds to lifting the lockdown in all the Region after the peak has fallen by 30%, while ${t}_{i}^{Async}$ corresponds to lifting the lockdown in the ${i}{\mathrm{th}}$ Region after the peak *of such Region* has fallen by 30%.

Starting with the first confirmed cases in Lombardy on 21th February, by the beginning of March the Covid-19 outbreak had already spread to the entire Italian territory. While the delay in the beginning of the infection is accounted for by the different mobility interactions between Italian Regions, once the epidemic has started in a given area, the intake of external infected people becomes quickly irrelevant. As a consequence, the normalised growth curves of the epidemic variables tend to converge to a similar shape if epidemiological parameters are uniform across the Regions. In fact, according to the Regional info-graphics released by the ISS^7^, Regional diffusion curves have a similar behavior but with different starting dates (see Fig. 4A,B). This observation can be justified as follows: Italian Regions are almost distinct administrative entities, where most of the population tend to work inside the resident Region^42^. Hence, the epidemic propagates from Region to Region via the fewer inter-Regional exchanges (incidentally, Lombardy is the Italian Region which is most involved in international trade connections^43^, being a natural candidate for the initial outbreak of the epidemic in Italy).

To test the effects of delayed epidemic starting dates, we build up a synthetic scenario where the Covid-19 outbreak spreads independently in each Region (see “Methods” for the estimation of the experimental time delays and for the Regional $SIOR$ model); as argued before, given the Italian mobility structure, such an approximation appears reasonable after the epidemic has started and is even more appropriate during the lockdown phase. Hence, we apply the parameters of Table 1 to Regional cases, applying System 1 separately to each Region. More specifically, the maximum number of individuals ${N}_{i}$ refers to the population of the *i*th Region^44^, while the initial times of the infection are assumed as those reported in Table 2, where we estimate the time delay by minimizing the distance among the observed curves. Notice that, assuming Lombardy as the first Region experiencing the virus contagion (i.e. delay = 0), the resulting Regional delays are mostly correlated to geographical distances from Lombardy.

**Table 2:** Regional delays (in days).

Epidemic delays across Italian Regions			
Lombardia	0.0	Molise	10.6
Emilia Romagna	3.1	Umbria	11.8
Marche	4.3	Abruzzo	13.1
Veneto	5.7	Lazio	14.5
Valle d’Aosta	6.4	Campania	15.0
P.A. Trento	6.6	Puglia	15.7
P.A. Bolzano	8.0	Sardegna	16.2
Liguria	8.1	Sicilia	16.6
Friuli Venezia Giulia	8.9	Calabria	17.2
Piemonte	9.0	Basilicata	19.2
Toscana	10.4		

The delay times are calculated by minimising the distance among the Regional counts of detected infections normalised by the population of the Region. Since Lombardy has been selected as the reference curve (hence, its delay is 0), the other Regions’ delays are calculated with respect to it.

By summing up all the ${S}_{i},\dots ,{R}_{i}$, respectively, we obtain a synthetic model for the global evolution of Covid-19 epidemic throughout Italy. To evaluate the effect of heterogeneity in time delays, we compare the number of daily cases ${O}^{\mathrm{Delay}}=\sum {O}_{i}^{\mathrm{Delay}}$ in our scenario (obtained by taking into account the Regional delays ${t}_{i}$ as reported in Table 2) with the number of daily cases ${O}^{0}=\sum {O}_{i}^{0}$ we would observe by considering the epidemic starting at the same time ${t}_{0}$ in all Regions. As expected, heterogeneity flattens the curve and shifts its maximum later in time. This is a first source of biases when fitting a heterogeneous dynamics with a global model. Again, we remark here that we are simply exploring realistic qualitative scenarios, without the aim to predict the real evolution of the epidemic: in fact, Italian Regions present relevant differences in terms of social contact habits, mobility flows, organization and capacity of health care provision, as well as for factors that affect the medical parameters, like comorbidities, social conditions or pollution levels, which should be taken into account to appropriately design the evolution of the epidemic.

As regards lockdown lifting scenarios, we consider two possible strategies: in the first, that we label the *Async*hronous scenario, each Region $i$ lifts the lockdown at the time ${t}_{i}^{\mathrm{Unlock}}$ when the peak of ${O}_{i}^{\mathrm{Delay}}$ decreases by 30%; in the second, that we label the *Sync*hronous scenario, each Region $i$ lifts the lockdown at the same time ${t}^{\mathrm{Unlock}}$, i.e., when the global peak of ${O}^{\mathrm{Delay}}$ decreases by 30%. The choice of 30% is arbitrary and similar results would hold for choices of values near the peak (see Fig. S6 in SI); it tries to be a sketch of a situation where, due to economic pressure, lockdown restrictions are lifted as soon as possible.

The epidemic dynamics in a given Region $i$ is essentially uncorrelated with the epidemic spreading of any other Region $j\ne i$ once the outbreak has started. For this reason, it could be appropriate to evaluate the lockdown lifting time on a Regional basis rather than lifting restrictions at the same time across each Region. Indeed, it could appear unreasonable to keep locked those Regions where the epidemic started earlier; on the contrary, Regions where the epidemic began with some delay could experience a strong rebound when subjected to a premature lockdown lifting. For instance, Fig. 4, panel C and D, shows the effects of lifting the lockdown at both Regional (*Async*) and national (*Sync*) level for two representative Italian Regions, namely Lazio and Lombardy. Since not only the epidemic, but also economy backslashes are non-linear processes, the *Sync* scenario can turn out to be even more disruptive than the epidemic itself (see also Fig. 3 and Table S1 in SI). Notice that analogous arguments hold—mutatis mutandis—also for the world/countries scenario as long as no super-spreaders^45^ change the probability of conveying abroad the epidemic.

## The role of age

As we have already observed in the previous Section, heterogeneity strongly impacts on the estimation of the results of the model^46^. Since the transmission coefficient is proportional to the contact rate between individuals, the rates of social mixing between different age classes represent a well known important source of heterogeneity within the model. This information can be estimated either through large-scales surveys^41^ or through virtual populations modeling^47^. While POLYMOD^41^ matrices have been extensively employed to estimate the cost-effectiveness of vaccination for different age-classes during the 2009 H1N1 pandemic^48,49^, here we use such information to support the design of a broad class of exit strategies. Hence, to account for age classes, we extend our model by rewriting the transmission coefficient as $\beta C$ (see “Methods” for a full description of the extended model), where $\beta$ is the transmission probability of the infection, and $C$ is the matrix describing the contact patterns typical of a given country. Because of lack of further information, we assume $\beta$ constant among age classes and $C$ as in Ref.^41^. To simplify the analysis, we gather POLYMOD age groups into three different classes: $Y$oung ($00{-}19$), $M$iddle ($20{-}69$) and $E$lderly (70+) (see Table 3). Such an aggregation combines the most “contactful” classes ($00{-}19$), the classes with the highest mortality risk (70+)^7^, and—with a good approximation—the classes corresponding to the active working population ($20{-}69$).

**Table 3:** POLYMOD matrix aggregated for three age classes: $Y$oung ($00{-}19$), $M$iddle ($20{-}69$) and $E$lderly (70+).

	Y	M	E
Y	2.35	0.44	0.67
M	0.47	0.59	0.50
E	0.50	0.55	0.80

Notice that $Y$ has the highest self-contact rate, followed by $E$ and then by $M$.

**Figure 5:**
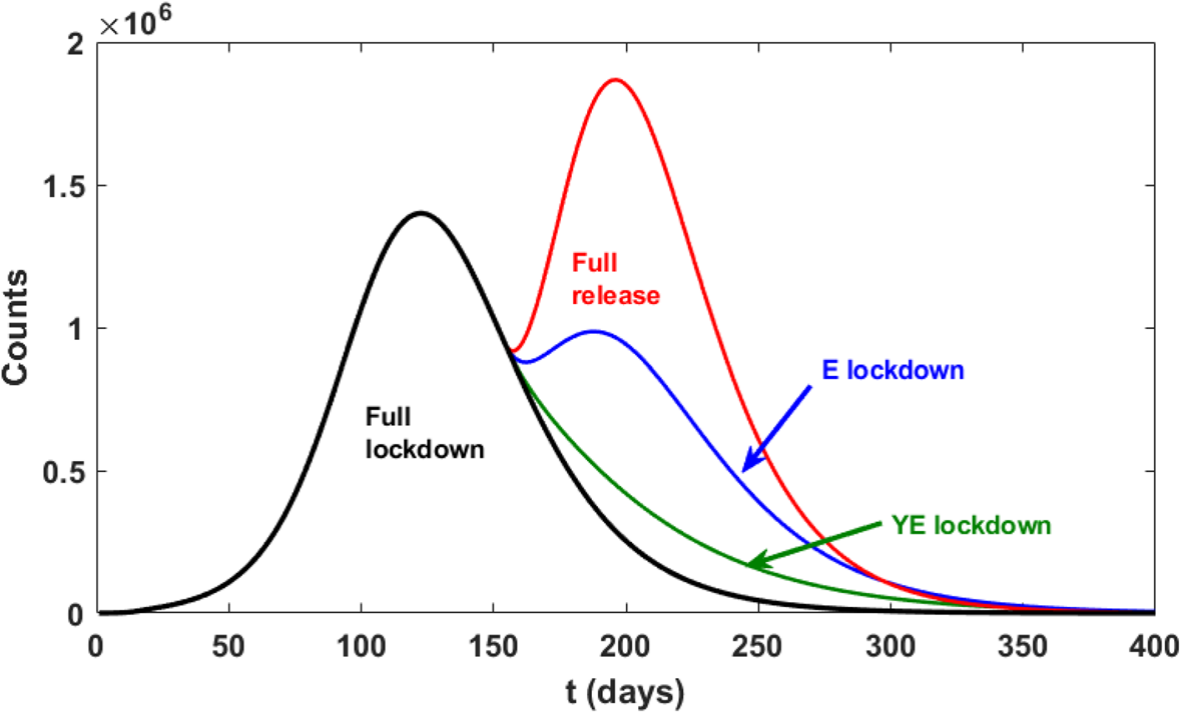
Comparison of the scenarios where the lockdown is relaxed only for a particular age class with respect to a full release policy. Strategies: YE = quarantine young and elderly, E = quarantine elderly. Notice that we have purposefully left the M class fully unrestrained in order to show how maintaining a partial, age-based lockdown could deeply change the effectiveness of the exit strategy, while maintaining active working force active.

Figure 5 shows how the number of people with observed symptoms ($O$) varies once the age class heterogeneity is considered in the model. Differently from Fig. 3, fully lifting the lockdown results in a second wave of the epidemic, where we observe a higher number of infected cases with respect to the homogeneous case. These findings highlight the importance of explicitly considering this source of heterogeneity in epidemic models for preventing underestimations of lockdown lifting consequences.

To estimate the importance of the age classes, we simulate mock-up lockdown release scenarios where some age classes are kept under lockdown even after the release day. As an example, in an E scenario, the contact rate of class E among itself and with other classes is damped by a factor $\alpha$ while the contact rates of Y and M among themselves returns to the pre-lockdown values. Results are shown in Fig. 5 together with the “full release” (i.e. no dampening of the contact matrix) strategy (see also Table S2 in SI).

Hence, the introduction of the age structure in the model allows us to guide the design of exit strategies based on age-targeted policies, as a way to dampen a possible upturn of contagion. Specifically, social/physical distance measures applied to the elderly may contribute to contain the impact of a renewed upward phase, while relaxing restrictions to the working age class (20–69) would not impair the smoothing of contagion propagation in the post-lockdown phase. Moreover, different strategies would change the relative percentages of age classes who have undergone the infection; since the incidence of severe cases is strongly age-related, this is a crucial issue. Again, it is important to emphasize that we are referring to simple mock-up strategies, which correspond to worst-case scenarios: in real life, community measures and physical distancing, infection prevention and control, personal hygiene habits, face mask usage, etc. will be decisive in contributing to dampening the epidemic^50^.

## Conclusions

In this paper we propose and test a general framework to study the Covid-19 contagion through a compartmental model, with a focus on geographical groups and age classes. Our framework shows that the promptness of lockdown measures has a main effect on the timing of the contagion. Strict social distancing policies reduce the severity of the epidemic during the lockdown period, but a full recover of the contagion can occur once such measures are relaxed. As a consequence, a mix of specific mitigation strategies must be prepared during the lockdown and implemented thereafter. In order to understand the relative potential impact of different strategies, we focus on two broad decomposition criteria within the model, i.e. on geographical mobility and on social interactions between age classes. First, we show how local dynamics at Regional level can be erroneously masked when observing the aggregate national system. Regional heterogeneity tends to lower and widen the curve of the contagion, contributing to shifting forward in time the epidemic peak at the aggregate level. Second, our analysis of mobility data shows that, due to the strong sparsity of interconnections across Regions, contagion develops independently within each Region once the epidemic has started. This, in turn, contributes to account for the delays observed in the alignment of the contagion curves across different geographical areas. The independence of Regional dynamics is important, since it could justify the adoption of a mix between general mitigation strategies and solutions which are specific to individual Regions (or countries) or clusters of Regions. Finally, we investigate the structure of social contacts across different settings and we quantify the relative importance of interactions between age classes in the spreading of contagion. We show that the younger (0–19) and the elder (70+) are the most intensively interacting classes. As a consequence, mitigation strategies specific to behaviours and interactions of individuals belonging to these two classes can produce a significant impact on diffusion rates in the post-lockdown phase. Our results show the importance of implementing local preventive and physical distancing measures specific to the elderly, while providing information, rules and guidelines on behaviours to be adopted in social interactions, aimed to reduce the risk of contagion for the younger. Overall, our results provide some guidance on how to lift some of the restrictions on mobility for the active population (20–69), while smoothing and lessening the propagation of contagion in the post-lockdown phase.

Although our study is tuned on the Italian Covid-19 outbreak, our modeling approach is general enough to help us understand the role of relevant dimensions, beside the medical and pharmaceutical ones, in identifying the relative importance of different strategies introduced to contain the epidemic and to mitigate its effects. Our framework can contribute to mitigate the strength of the trade-off between health and economic outcomes. In particular, we show how the timeline of post-lockdown measures should take into account some fundamental compartmental aspects, such as geographical factors and the intensity and frequency of interactions between members of different age classes in different settings. This feature is general, and it can drive the analysis towards fine grained simulations on the impact of specific precautionary interventions, which enforce social distancing acting on proximity/local behaviours while containing the overall burden for the economy and society.

## Methods

The proposed $SIOR$ model belongs to the classic family of compartmental models^20^. As the most renewed $SIR$ and $SEIR$ (and their variations), it models the infection rate to be proportional to the number of individuals in a $S$(usceptible) compartment (i.e., the ones that have never been infected) times the probability of meeting infected people (modelled as the fraction $I/N$ of $I$(nfective) individuals respect to the population size $N$). The rate indicating individuals that are $R$emoved from the $I$ class, either because recovered and no more susceptible or because deceased, is proportional to the number of individuals in $I$. To have the possibility of calibrating our model’s parameters with the observed data, we introduce another class $O$ of “observable” individuals, i.e. people with symptoms strong enough to be detected by the national healthcare system.

### Initial parameters estimation

In the early phases of the epidemic, the observed quantities follow an approximately exponential growth ${Y}^{\mathrm{Obs}}\sim {Y}_{0}{e}^{gt}$, as expected in most epidemic models. To understand their implications in our model, we notice that for $I/S\ll 1$ we can linearize System 1 resulting in $I\sim {I}_{0}{e}^{(\beta -\gamma )t}$ and $O\sim \rho \gamma I$. Thus, minimizing the difference between $O$ and ${Y}^{\mathrm{Obs}}$ in the early period would yield the estimates for $\beta ,\gamma$ such that $\beta -\gamma \sim g$, and ${R}_{0}\sim 1+g/\gamma$ would increase linearly with the characteristic time ${\tau }_{I}={\gamma }^{-1}$. Note that most of the compartmental models based on a set of ordinary differential equations show an initial exponential growth phase with the same exponent; hence, in the early stage of the epidemic, it is possible to successfully fit the “wrong” variables.

To adapt the $SIOR$’s parameters to the Italian data^7^, we compare the reported cumulative number of Covid-19 cases ${Y}^{\mathrm{Obs}}$ with the analogous quantity ${Y}^{\mathrm{model}}=\int \rho \gamma Idt$ in our model (see Fig. S2 in SI). We want to stress that our model fitting is not aimed to produce an accurate model for detailed predictions, but to work in a realistic Region of the parameters space. We estimate model’s parameters by least square fitting on the pre-lockdown period. Since in such range the data ${Y}^{\mathrm{Obs}}$ show an exponential growth trend, this indicates that the pre-lockdown period is an early phase of the epidemic where $\beta -\gamma$ equals the growth rate of ${Y}^{\mathrm{Obs}}$. For fixed $\beta -\gamma$, the time of the epidemic start (that we conventionally assume as the time ${t}_{0}$ where the number of infected is $1$) and the fraction $\rho$ of serious cases observed by the national healthcare System, allow some flexibility in estimating the values of $\beta$ and $\gamma$ as long as their difference is fixed. Hence, estimating *medical* parameters as the rate $\gamma$ of escaping the infected state is paramount for calibrating the model.

In response to the outbreak of Covid-19, several estimates of model parameters have been proposed in the literature, revealing a certain amount of uncertainty about some fundamental variables of the epidemic contagion. The European Centre for Disease controls reports an infection time duration ${\tau }_{I}$ between $5$ and $14$ days^50^; in our model, we will use ${\tau }_{I}=10$ (i.e. $\gamma ={\tau }_{I}^{-1}=1/10 \;\text{days}^{-1}$). According to a report of ISS^51^, the Italian National Health Institute, the time from the start of serious symptoms (i.e. when an infected individual results “observed” from ISS) to the resolution of the symptoms can be estimated as ${\tau }_{H}\sim 9 \;\text{days}$, corresponding, in our model, to a value $h=1/9 \;\text{days}^{-1}$. Notice that the analysis of 12 different models^38^ reports varying estimates for the basic reproduction number ${R}_{0}$, ranging from $1.5$ to $6.47$, with mean $3.28$ and a median of $2.79$.

From fitting the $15$ days of ${Y}^{obs}$ (pre-lockdown phase) and by performing a bootstrap sensitivity analysis of the parameters, we obtain $\beta -\gamma \sim 0.25\pm 0.01$ and ${t}_{0}=-30\pm 5 \;\text{days}$ by assuming $\rho =40{\%}$. Varying $\rho$ in $[10{\%},100{\%}]$ leads $\beta -\gamma$ in $[\mathrm{0.22,0.27}]$. On the other hand, for fixed $\beta -\gamma$, ${R}_{0}$ would vary linearly with ${\tau }_{I}$; as an example, ${R}_{0}$ varies in $[\mathrm{2.5,4.5}]$ for the literature parameters ${\tau }_{I}\in [\mathrm{5,14}]$; accordingly, to adjust the difference in the growth rate, ${t}_{0}$ varies in $[\mathrm{26,32}]$. However, despite the variability of the parameters’ range, the qualitative behavior of the model—and hence our analysis of the key factors of the epidemic evolution—is unchanged.

### Estimation of the experimental regional time delays

We first normalize the observed data by dividing the number of non-zero observations in a Region for its population. Let ${y}_{i}$ be the normalized observations for the ${i}^{\mathrm{th}}$ Region. For each pair of Regions $i,j$, we define the variation interval ${\Delta }_{ij}=[{\mathrm{min}}_{ij},{\mathrm{max}}_{ij}]$ that contains the maximum number of points of both ${y}_{i}$ and ${y}_{j}$, i.e. ${\mathrm{min}}_{ij}=\mathrm{max}\{\mathrm{min}({y}_{i}),\mathrm{min}({y}_{j})\}$ and ${\mathrm{max}}_{ij}=\mathrm{min}\{\mathrm{max}({y}_{i}),\mathrm{max}({y}_{j})\}$. The delay ${t}_{ij}$ between the epidemic start in $i$ and $j$, respectively, is computed by minimizing $\parallel ({\Delta }_{ij}\cap {y}_{i}(t))-({\Delta }_{ij}\cap {y}_{j}(t-{t}_{ij}){\parallel }_{2}$, where ${\Delta }_{ij}\cap y$ denotes the values of $y$ falling in the interval ${\Delta }_{ij}$ and $\parallel \cdot {\parallel }_{2}$ denotes the quadratic norm. Denoting with ${T}_{i}$ the times corresponding to the observation in ${\Delta }_{ij}\cap {y}_{i}$, it is easy to verify that ${t}_{ij}=\langle {T}_{i}\rangle -\langle {T}_{j}\rangle$, where $\langle T\rangle$ is the average value of the times contained in $T$.

### Equivalence of normalized curves

System 1 referred to Region $k$ becomes:

3 $$\begin{array}{ll}{\partial }_{t}{S}_{k}& =-\beta {S}_{k} {I}_{k}/{N}_{k}\\ {\partial }_{t}{I}_{k}& =\beta {S}_{k} {I}_{k}/{N}_{k}-\gamma {I}_{k}\\ {\partial }_{t}{O}_{k}& =\rho \gamma {I}_{k}-h{O}_{k}\\ {\partial }_{t}{R}_{k}& =(1-\rho )\gamma {I}_{k}+h{O}_{k}\end{array}$$

where ${N}_{k}$ is the population size of $k$. By rewriting System (3) in terms of normalized quantities ${s}_{k}={S}_{k}/{N}_{k},\dots {s}_{k}={S}_{k}/{N}_{k}$, we obtain the same set of equations for all the Regions:

4 $$\begin{array}{ll}{\partial }_{t}s& =-\beta s i\\ {\partial }_{t}i& =\beta s i-\gamma i\\ {\partial }_{t}o& =\rho \gamma i-ho\\ {\partial }_{t}r& =(1-\rho )\gamma i+ho\end{array}$$

Hence, for similar initial conditions, by normalizing the experimental observations by the population size, one should obtain similar epidemic dynamics if the Regional parameters are the same. Notice that, while parameters like $\gamma$ are not expected to vary on Regional bases, $\beta$ is likely to vary since it reflects differences in social interactions. The same reasoning applies to countries or to large, independent administrative units like metropolitan areas or megacities.

### Regional SIOR model

Let us assume of knowing the fraction ${Z}_{kl}$ of people commuting from Region $k$ to Region $l$. Then, System (4) becomes:

5 $$\begin{array}{ll}{\partial }_{t}{s}_{k}& =-\beta {s}_{k} \sum_{l}{Z}_{kl} {i}_{l}\\ {\partial }_{t}{i}_{k}& =\beta {s}_{k} \sum_{l}{Z}_{kl} {i}_{l}-\gamma {i}_{k}\\ {\partial }_{t}{o}_{k}& =\rho \gamma {i}_{k}-h {o}_{k}\\ {\partial }_{t}{r}_{k}& =(1-\rho )\gamma {i}_{k}+h {o}_{k}\end{array}$$

From mobility data, we know that ${\epsilon }_{k}={\sum }_{l\ne k}{Z}_{kl}/{Z}_{kk}\ll 1$ and ${Z}_{kk}\sim 1$; in particular, from Facebook mobility data we can estimate $\langle {\epsilon }_{k}\rangle \sim 1{0}^{-3}$. If all the neighbors of a given Region $k$ are fully infected (i.e. ${i}_{l}=1 \forall l\ne k$) and ${i}_{k}({t}_{0})=0$, then the variation of ${i}_{k}$ can be approximated as ${\partial }_{t}{i}_{k}\sim {\epsilon }_{k}+(\beta -\gamma ) {i}_{k}$. Namely, as soon as ${i}_{k}>{\epsilon }_{k}$, ${i}_{k}$ will grow exponentially according to ${\partial }_{t}{i}_{k}\sim (\beta -\gamma ) {i}_{k}$ and ${\epsilon }_{k}$ will become irrelevant, the epidemic dynamics of the Regions will decouple. On the other hand, if epidemic is decaying everywhere, then ${i}_{l}\ll 1 \forall l\ne k$; thus ${\sum }_{l\ne k}{Z}_{kl} {i}_{l}\ll {\epsilon }_{k}$ and the system again decouple, and the evolution of the epidemic in each Region will be described by the decoupled equations of System 4.

### Social mixing

To take account for social mixing, we rewrite the transmission coefficient as the product of a transmission probability $\beta$ times a contact matrix $C$ whose element ${C}_{ab}$ measure the average number of (physical) daily contacts among an individual in class age $a$ and an individual in class age $b$. Notice that the probability that a susceptible in class $a$ has a contact with an infected in class $b$ is the product of the contact rate ${C}_{ab}$ times the probability ${I}_{b}/{N}_{b}$ that individual in class $b$ is infected. Hence, denoting with ${S}^{a},\dots ,{R}^{a}$ the number of $S$(usceptibles),$\dots$,$R$(emoved) individuals in class age $a$, we can rewrite System (1) as:

6 $$\begin{array}{ll}{\partial }_{t}{S}^{a}& =-\beta {S}^{a}\sum_{b}{C}_{ab}\frac{{I}^{b}}{{N}_{b}}\\ {\partial }_{t}{I}^{a}& =\beta {S}^{a}\sum_{b}{C}_{ab}\frac{{I}^{b}}{{N}_{b}}-\gamma {I}^{a}\\ {\partial }_{t}{O}^{a}& =\rho \gamma {I}^{a}-h{O}^{a}\\ {\partial }_{t}{R}^{a}& =(1-\rho )\gamma {I}^{a}+h{O}^{a}\end{array}$$

Although the form of System (6) is similar to System (3), here it is not possible to consider a separate evolution for the different age classes since, differently from the inter-Regional mobility matrix $Z$, the off diagonal elements of the social matrix ${C}_{a,b},a\ne b$, are of the same order of the diagonal elements ${C}_{aa}$, i.e. interactions among different age classes are of the same magnitude of interactions among individuals of the same age class. Hence, the exogenous contribute of other classes $b\ne a$ to the infected growth rate ${\partial }_{t}{I}_{a}={\sum }_{b\ne a}{C}_{ab}{I}^{b}/{N}_{b}$ cannot be disregarded respect the endogenous contribute ${C}_{aa}{I}^{a}/{N}_{a}$. Obviously, we are not considering the unrealistic case ${N}_{a}\gg {N}_{b}$.

Notice that an equation for the evolution of the total population can be obtained by summing up System (6), obtaining an equation in $S={\sum }_{a}{S}_{a},\dots ,R={\sum }_{a}{R}_{a}$ of the form of System (1) but with age classes appearing in the re-normalized infection parameter $\beta \to \beta {C}^{\mathrm{eff}}$, where ${C}^{\mathrm{eff}}=\frac{{\sum }_{ab}{C}_{ab}{S}^{a}{I}^{b}/{N}_{b}}{SI/N}$ is the average contact value among infected and susceptible individuals of all age classes. Thus, not taking into account age classes would lead to measure a time-dependent ${R}_{0}$ even with time independent parameters.

## Supplementary information

Supplementary Information 1.
