# Supplementary material for: Time, space and social interactions: exit mechanisms for the Covid-19 epidemics
Source: Sci Rep. 2020 Aug 13;10:13764. doi: 10.1038/s41598-020-70631-9 (PMC7426873; doi:10.1038/s41598-020-70631-9)
Supplement: Supplementary file 1 — Supplementary Information 1. [file 41598_2020_70631_MOESM1_ESM.docx]

##### Supplementary information Time, Space and Social Interactions: Exit Mechanisms for the Covid-19 Epidemics

Antonio Scala^1,2,3*^, Andrea Flori^4^, Alessandro Spelta^5^, Emanuele Brugnoli^1^, Matteo Cinelli^1^, Walter Quattrociocchi^7,1^, Fabio Pammolli^4,6^

Sat Jul 18 09:28:26 2020

[1]Applico Lab, CNR-ISC [2]Big Data in Health Society [3]Gubkin Russian State University of Oil and Gas, Leninsky Prospekt, Moskow [4]Impact, Department of Management, Economics and Industrial Engineering, Politecnico di Milano [5]Univ. di Pavia [6]Center for Analysis Decisions and Society, Human Technopole and Politecnico di Milano [7]Univ. di Venezia ’Ca Foscari

## 1 Supplementary Figures


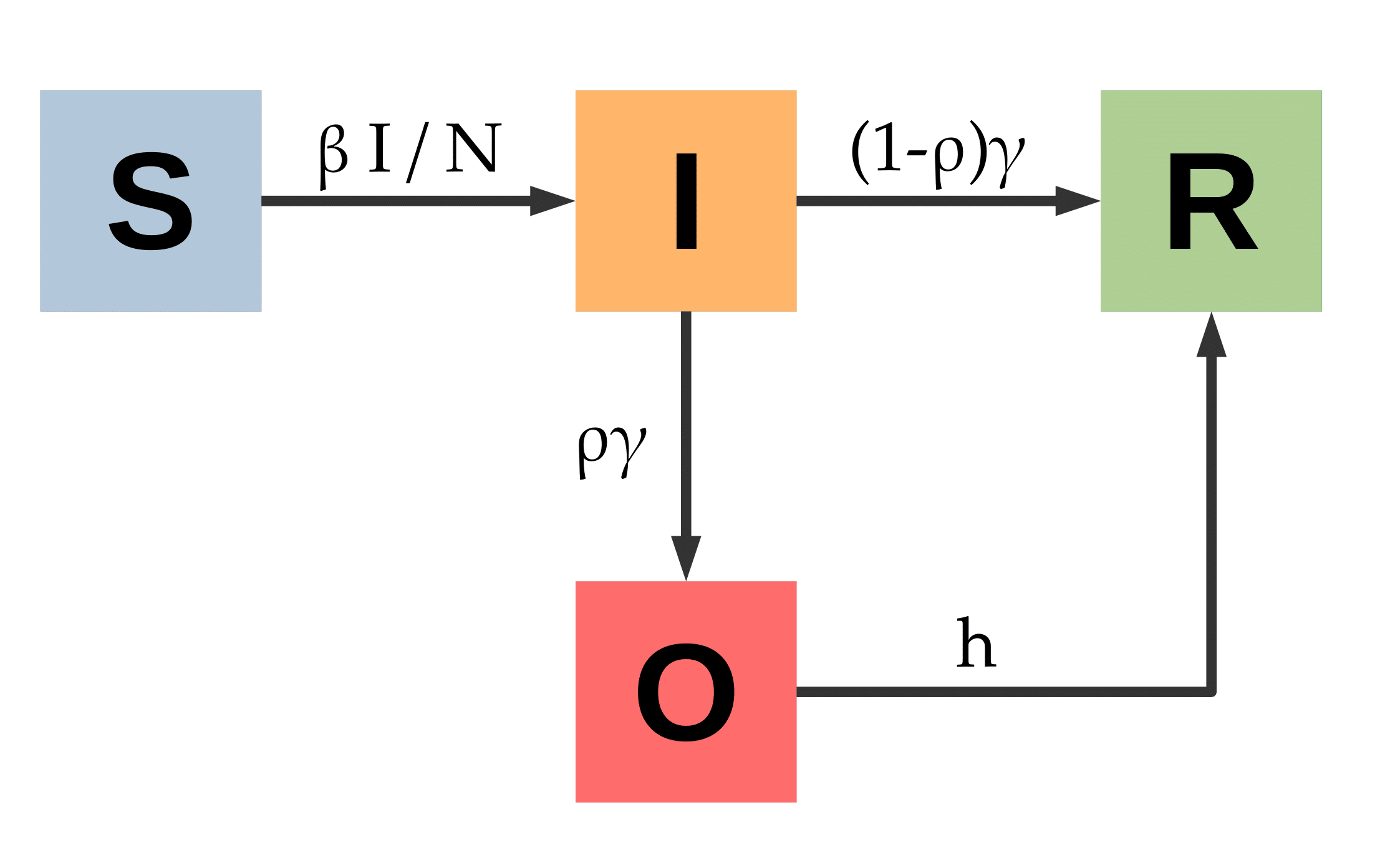


Supplementary Figure 1: The $SIOR$ compartmental model: workflow of the epidemic process. A $S$(usceptible) individual becomes $I$(nfective) when meeting an infective person. An $I$(nfective) either become $O$(bserved), with symptoms acute enough to be detected from the national health-care System, or is $R$(emoved) from the infection cycle by having recovered. An $O$(bserved) individual can also be $R$(emoved) from the infection cycle having become immune. The parameter $\beta$ defines the rate at which a susceptible becomes infectious, $\gamma$ represents the rate at which infectious either become observable or recover, $\rho$ is the fraction of infectious that become observed from the national health-care System and $h$ is the rate at which observed individuals are removed from the infection cycle.


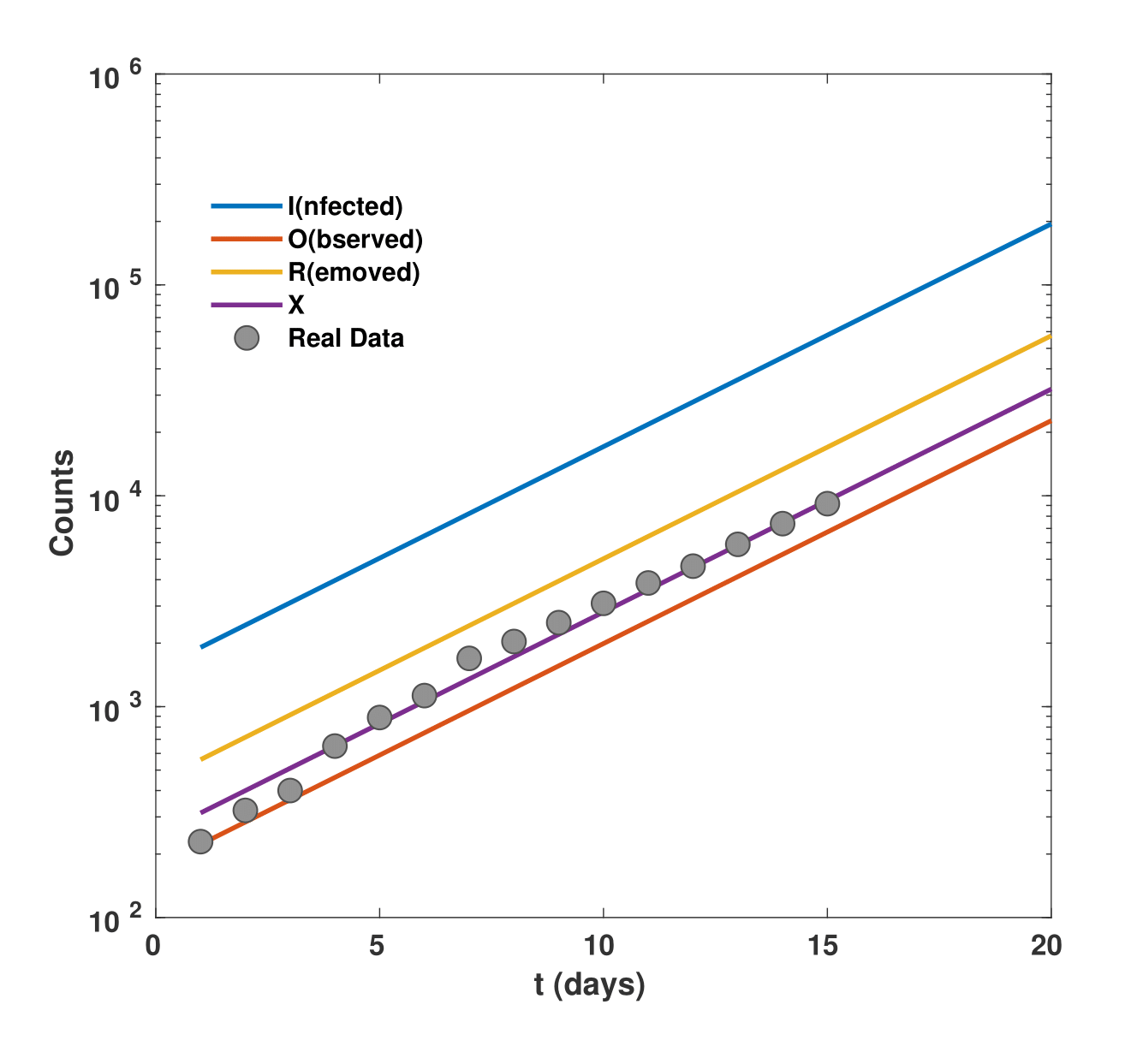


Supplementary Figure 2: In the initial stage, most of the quantities experience an exponential growth with the same exponent; hence, it would be possibly to “successfully” fit the wrong variables. The Figure shows the pre-lockdown growth of the number of $I$(nfected), $O$(bserved), $R$(emoved) individuals in our model. Full circles represent the experimental counts of confirmed Covid-19 cases in Italy; $X$ is the cumulative variable we use to fit the experimental data.


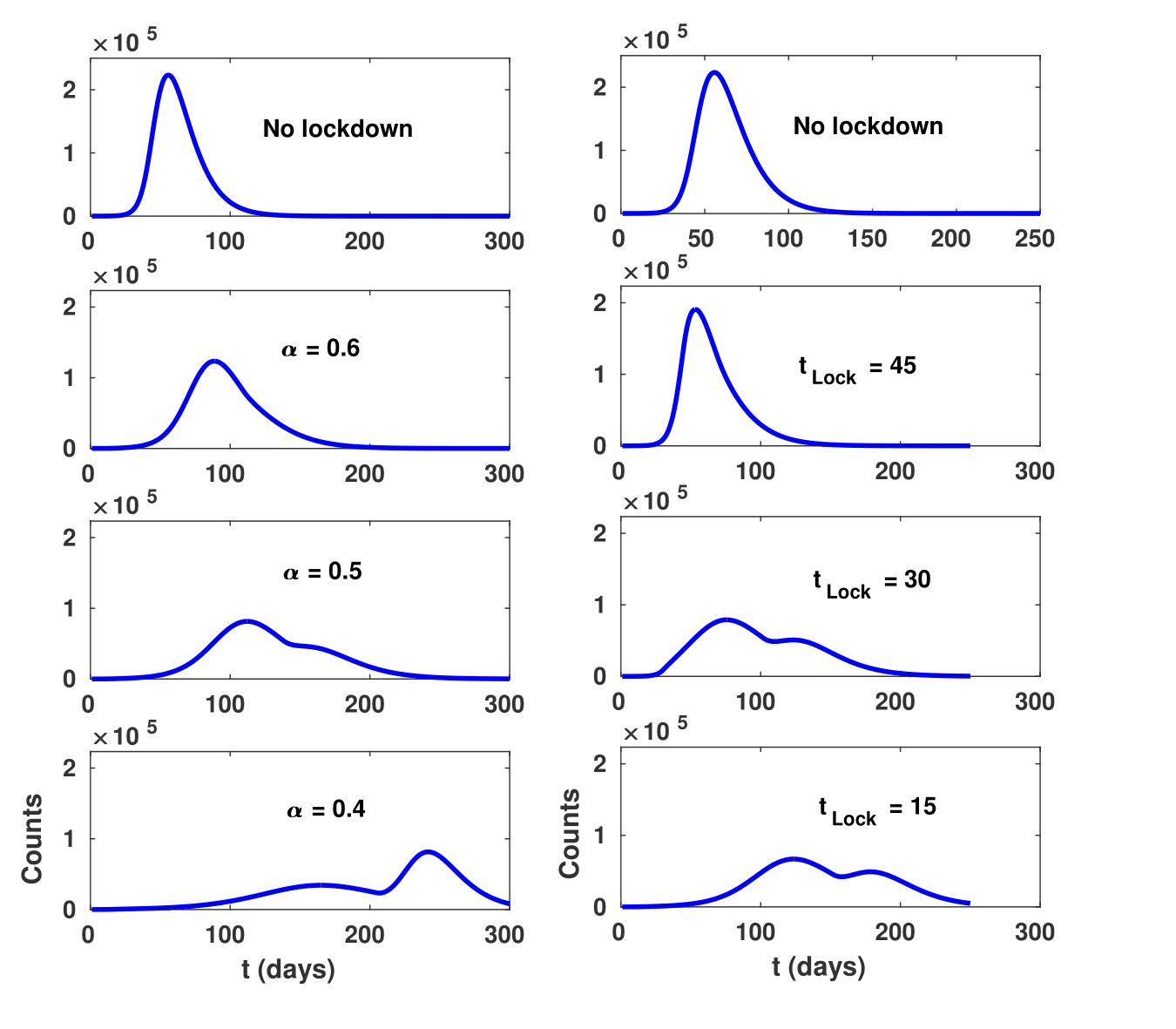


Supplementary Figure 3: Left panel: variation of the behavior of the model by varying the lockdown strength $\alpha$. Lockdown starts at $t_{\mathrm{Lock}}=15$ and is fully lifted when the peak has fallen by $30\%$. Right panel: variation of the behavior of the model by delaying the lockdown starting time $t_{\mathrm{Lock}}$. Lockdown strength is fixed at $\alpha=0.5$ and is fully lifted when the peak has fallen by $30\%$.


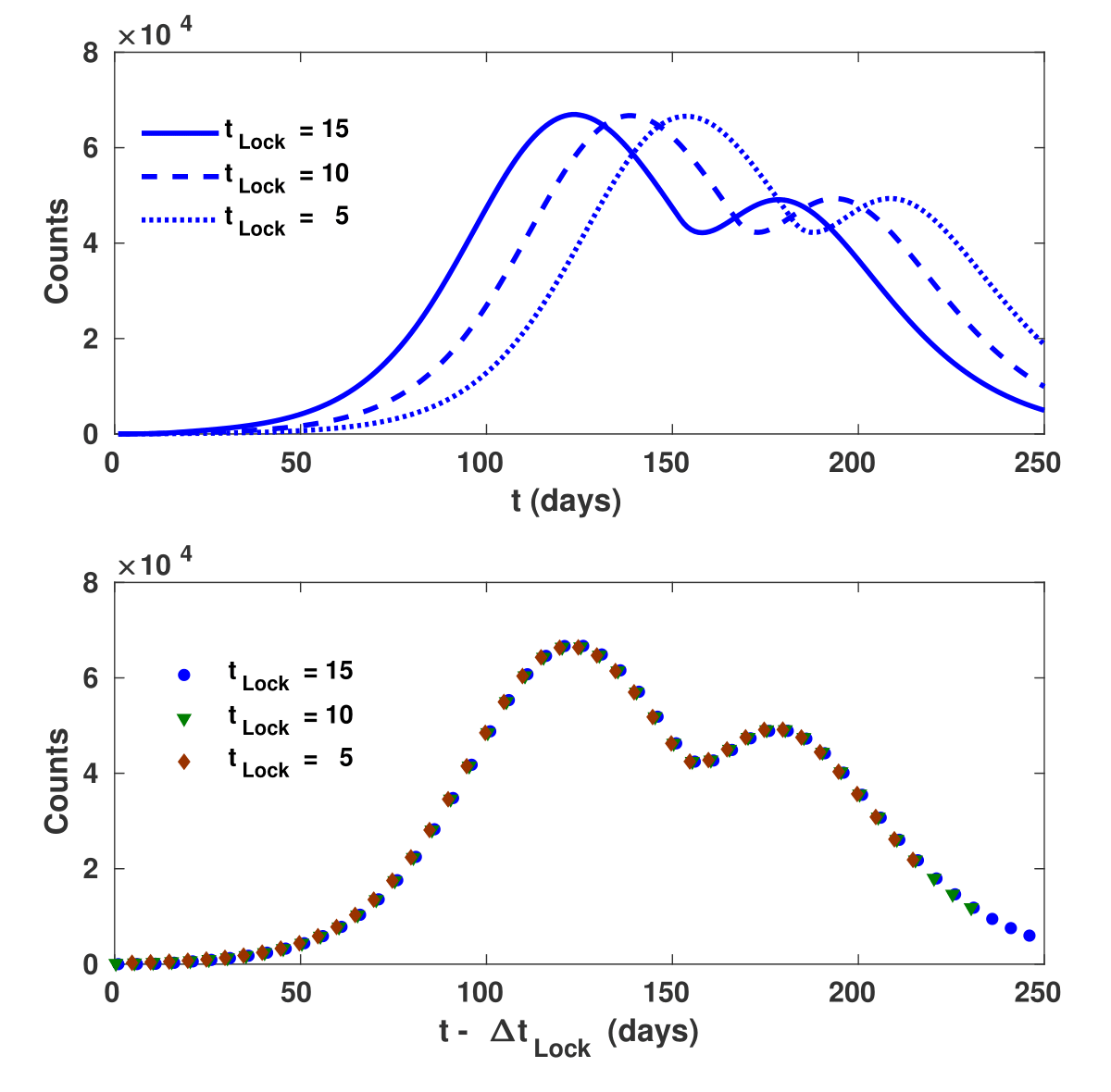


Supplementary Figure 4: Upper panel: variation of the behavior of the model by anticipating the lockdown time. Notice that anticipating the lockdown leaves unchanged the behaviour of the epidemics, just shifting all the times of an amount proportional to how much the lockdown is anticipated. Lockdown strength is fixed at $\alpha=0.5$ and is fully lifted when the peak has fallen by $30\%$. Lower panel: by applying the Eq. 2, we show how the curves in the upper panel collapse on each other.


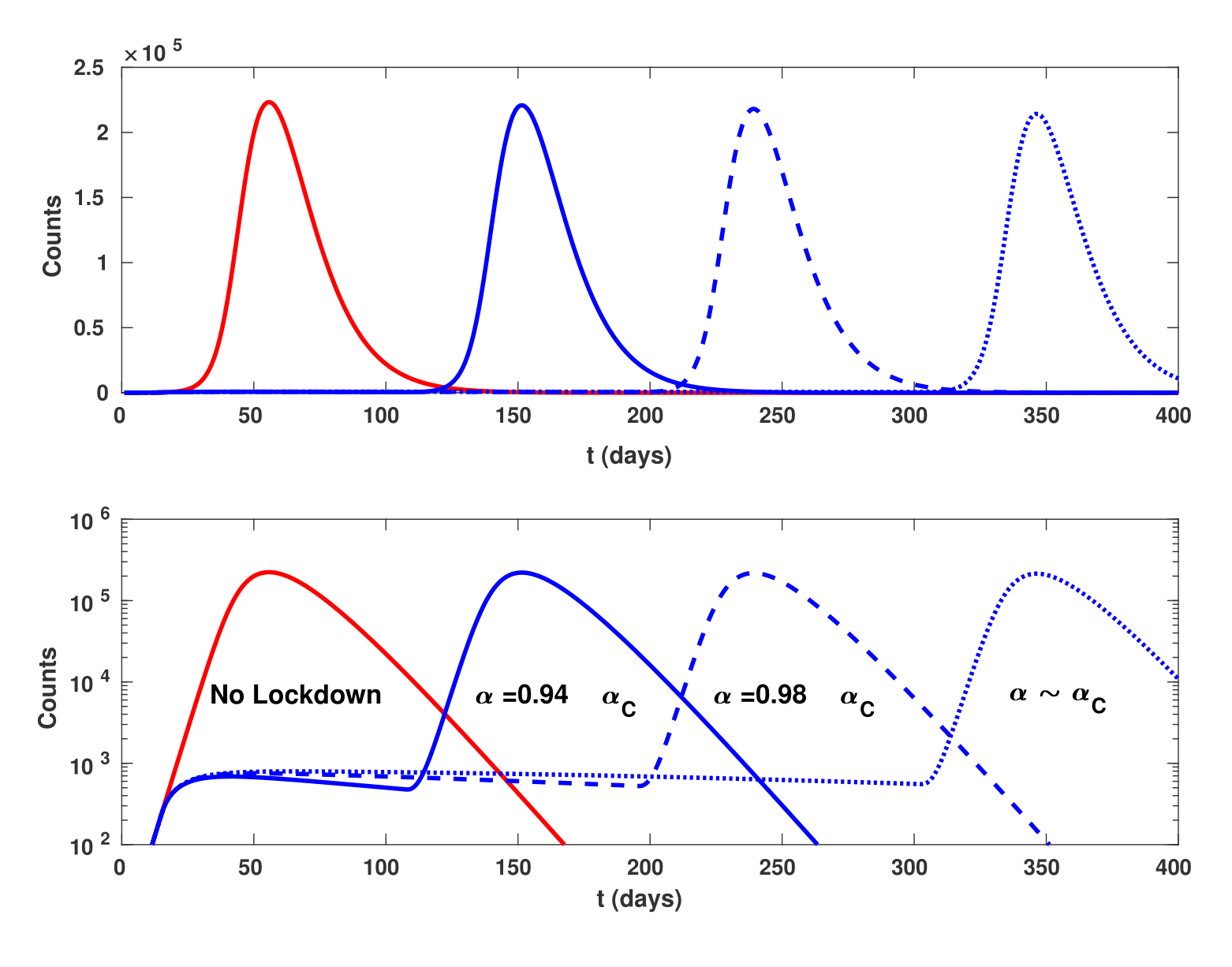


Supplementary Figure 5: Upper panel: variation of the behavior of the model for lockdown strengths $\alpha<\alpha_{\mathrm{crit}}=1/R_{0}$. Notice that the height of the peaks after the lockdown lifting is almost unchanged if compared with the no lockdown scenario. Lockdown starting date is fixed at $t_{\mathrm{Lock}}=15$ and is fully lifted when the peak has fallen by $30\%$. Lower panel: for better clarity, the plot is also reported in log-linear scale.


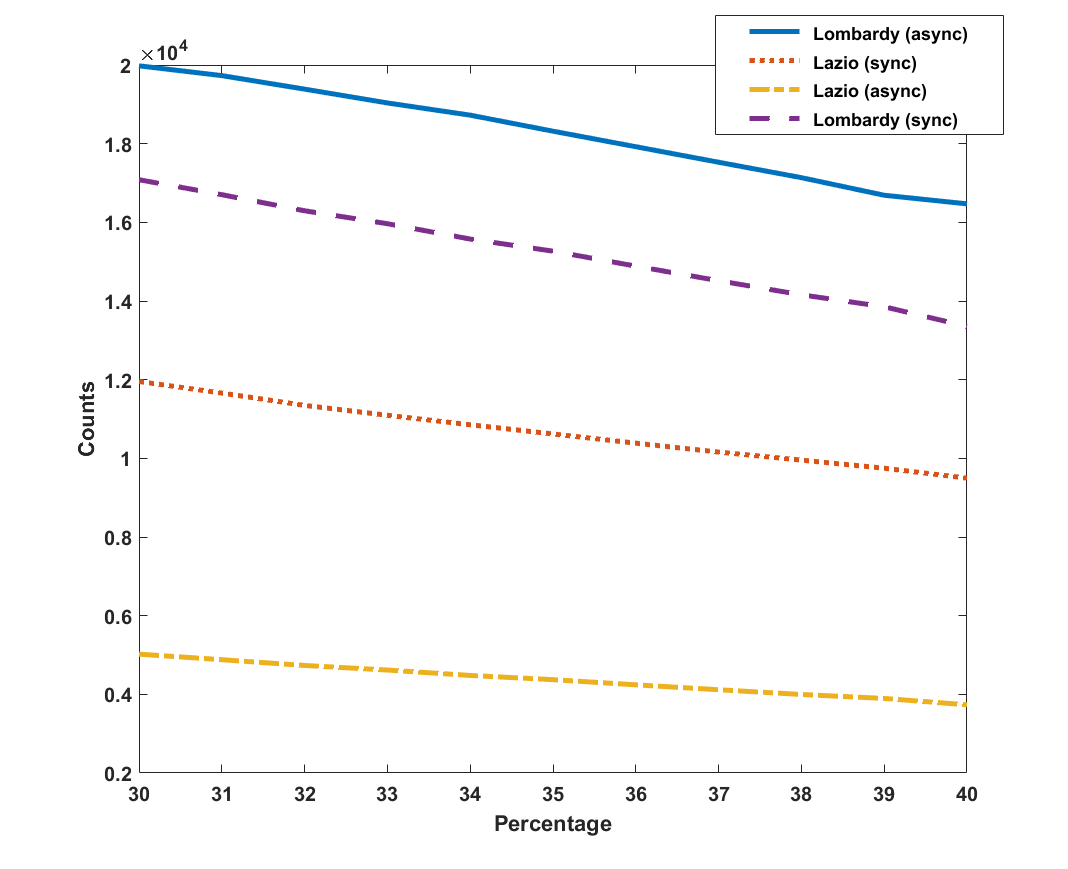


Supplementary Figure 6: Sensibility analysis of the SYNC and ASYNC scenarios reported in the paper. On the y-axis it is reported the height of the second peak after the lockdown release; on the x-axis the percentage of the drop at which lockdown is released. As an example, a percentage of $35\%$ means that lockdown is released when the counts of O(bserved) has fallen by $35\%$ of its peak value. As shown in the figure, waiting more to release the lockdown (i.e. releasing at higher percentages) lowers the second peak. In our case, switching the threshold to release the threshold from $30\%$ to $40\%$ reduces the height of the second peak by $\sim20\%$; on the other hand, the second peak shifts in time by a factor that varies from $\sim2\%$ to $\sim9\%$ in the scenarios of the figure

## 2 Supplementary Tables

|  | Lom-A | Laz-S | Laz-A | Lom-S |
| --- | --- | --- | --- | --- |
| $\Delta Max2/Max2$ | 17.6% | 20.6% | 25.6% | 21.8% |
| $\Delta t_{Max2}/t_{Max2}$ | 2.3% | 2.8% | 8.9% | 4.2% |

Table 1: Relative variations of the counts for the second maximum ($Max2$) and its occurrence ($t_{Max2}$) when the lockdown is released after a decrease of $30\%$ or of a $40\%$ from the peak. The scenarios are indicated by the first three letters of the region (Lom=Lombardy, Laz=Lazio) and by A or S to indicate an Asynchronous or a Synchronous release of the lockdown over the Italian territory.

| **(a)** | Y | M | E |
| --- | --- | --- | --- |
| YE | 63% | 71% | 72% |
| E | 16% | 40% | 54% |
| Y | 42% | 27% | 16% |

| **(b)** | Y | M | E |
| --- | --- | --- | --- |
| YE | 69% | 76% | 76% |
| E | 21% | 46% | 59% |
| Y | 46% | 31% | 20% |

Table 2: Performance of the age-class lockdown strategies. Columns are the age classes: Y for young (0-19 years), M for middle (20-69 years), E for elderly (70+ years). Rows are the strategies: YE indicates that Young and Elderly are segregated, E that Elderly are segregated, Y that Young are segregated. Table entries represent the relative gain in terms of cumulative infected respect the full lockdown release. As an example, strategy E lowers the number of elderly infected by $\sim50\%$ but lowers the number of young that get infected by only $\sim15\%$. For these tables, it is assumed that the contact rate for segregated people stays at $\alpha\sim0.49$ as during the lockdown; the release of the lockdown happens when the O(bserved) counts drops to $30\%$ of its peak (left table (a)) or to $50\%$ of its peak (right table (b)).
